# Improved accuracy of S-value-based dosimetry: a guide to transition from Cristy–Eckerman to ICRP adult phantoms

**DOI:** 10.1186/s40658-022-00485-9

**Published:** 2022-08-26

**Authors:** Shalini Subramanian, Bin He, Eric Frey, Derek W. Jokisch, Wesley Bolch, George Sgouros

**Affiliations:** 1 Radiopharmaceutical Imaging and Dosimetry, LLC (Rapid), Baltimore, MD USA; 2 The Russell H. Morgan Department of Radiology and Radiological Science, School of Medicine, Johns Hopkins University, Baltimore, MD USA; 3 Department of Physics and Engineering, Francis Marion University, Florence, SC USA; 4 Center for Radiation Protection Knowledge, Oak Ridge National Laboratory, Oak Ridge, TN USA; 5 J. Crayton Pruitt Family Department of Biomedical Engineering, University of Florida, Gainesville, FL USA

**Keywords:** ICRP phantom, Radiopharmaceutical dosimetry, Cristy–Eckerman phantom

## Abstract

**Background:**

In 2016, the International Commission on Radiological Protection (ICRP) published the results of Monte Carlo simulations performed using updated and anatomically realistic voxelized phantoms. The resulting specific absorbed fractions are based on more realistic human anatomy than those computed in the stylized, geometrical Cristy–Eckerman (CE) phantom. Despite this development, the ICRP-absorbed fractions have not been widely adopted for radiopharmaceutical dosimetry. To help make the transition, we have established a correspondence between source and target tissues defined in the CE phantom and those defined in the ICRP phantoms.

**Results:**

The ICRP phantom has 79 source regions and 43 target regions in comparison with the 23 source and 18 target tissue regions defined in the CE phantom. The ICRP phantom provides tissue regions with greater anatomical detail. Some of this additional detail is focused on radiation protection and dosimetry of inhaled/ingested radioactivity. Some, but not all, of this detail is useful and appropriate for radiopharmaceutical therapy. We have established the correspondence between CE and ICRP phantom source and target regions and attempted to highlight the ICRP source tissues relevant to radiopharmaceutical therapy (RPT). This paper provides tables and figures highlighting the correspondences established.

**Conclusion:**

The results provide assistance in transitioning from CE-stylized phantoms to the anatomically accurate voxelized ICRP phantoms. It provides specific guidance for porting the total absorbed activity for regions as defined in the CE phantom to regions within the ICRP phantoms.

## Introduction

“Be not the first by whom the new are tried

Nor yet the last to lay the old aside”

(Alexander Pope)

The Medical Internal Radiation Dose Committee published MIRD Pamphlet No. 1 in 1968. This Pamphlet, along with Pamphlet Numbers 5 [1] and 11 [2], introduced the dosimetry formalism for internally distributed radionuclides [3]. The most recent update to this formalism [4] maintains the fundamental innovation of the first series of pamphlets. The radionuclide S value embodies the key innovation of the MIRD formalism over previous methods [5]. The S value defines the absorbed dose to a target tissue per unit transformation of the radionuclide in a source tissue, and as such, it depends upon the emission properties of the radionuclide and the source–target tissue anatomy (i.e., their shape, relative distance, and elemental composition as well as the composition of all tissues in between). The source–target tissue anatomy (or reference phantom) of the Cristy and Eckerman (CE) phantom set [6, 7] was used to calculate S values, as implemented in the widely used OLINDA/EXM software package that is still in use today. Consistent with the computational capabilities of the time, the CE phantom series represents organs as simple geometrical shapes or combinations thereof. Eighteen distinct tissues are represented, along with a general category, “other tissues,” which represents residual tissues of radiopharmaceutical uptake not already identified explicitly in the source tissues.

The International Commission on Radiological Protection (ICRP) has worked on generating updated and realistic voxelized phantoms [9]. In these phantoms, source and target regions are defined by segmentation in computed tomography (CT) data collected across patients and transformed to match representative organ masses using voxel-based scaling. A total of 79 distinct source regions along with 43 target regions were defined in these new phantoms. Note that there is overlap between some of the 79 regions as discussed further in the Results section. The greater accuracy resulting from the use of these new phantoms is essential for emerging radiopharmaceutical therapy (RPT) applications of dosimetry. These updated phantoms were later used to calculate specific absorbed fraction (SAF) values for both electron and alpha-particle transport along with more accurate photon transport simulations [8] taking into consideration the enhanced computational abilities. This work provides guidance and a pathway to allow investigators to transition to the more anatomically accurate ICRP phantoms for RPT dosimetry applications.

## Methods

The data presented by Cristy and Eckerman in [6] and that by the ICRP in its Publications 89, 133, and 110 [8–10] extensively describe the phantom models. By scanning through this literature, we have established an anatomical correspondence between the source and target regions in the CE and ICRP phantom to identify differences resulting from mass and geometry updates. Additionally, the figures in the results section help represent the mapping performed to transition from CE phantom to ICRP phantom. We also highlight the additional source regions defined and included in these ICRP publications, especially those relevant to RPT applications.

## Results

In 2008, the ICRP established for the first time its own voxelized reference phantoms for the adult male and adult female [10]. These phantoms were based upon CT imaging of height–weight matched patients and subsequently altered to specifically match reference values of organ mass given in ICRP Publication 89 [9]. The Publication 110 reference phantoms provide a detailed description of the human anatomy for a total of 79 source regions and 43 target regions [10].

The updated ICRP phantom regions reflect the ICRP’s focus on radiation protection. Accordingly, a number of new tissues and related anatomical details are intended to support organ dosimetry associated with inhaled or ingested radionuclides and their concomitant risks of radiation-induced stochastic bioeffects (e.g., cancer and hereditary effects). Although some of these regions may also be relevant source or target regions for radiopharmaceutical therapy, in certain circumstances a number of them are not. The respiratory tract and alimentary tract [12] models illustrate this difference in dosimetric purposes. The expanded versions of these two anatomical systems constitute more than half of the source regions listed in ICRP revised publications.

### ICRP respiratory tract model

To study the effects of inhaled radioactivity, the morphological model of the respiratory tract is divided into five tissue regions associated with deposited radioactive aerosols along the walls of the respiratory airways [11]. These include ET1 (anterior nasal cavity), ET2 (posterior nasal cavity), BB (bronchial airways), bb (bronchiolar airways), and AI (alveolar interstitial region), which collectively define the human respiratory tract model (or HRTM).

**Fig. 1:**
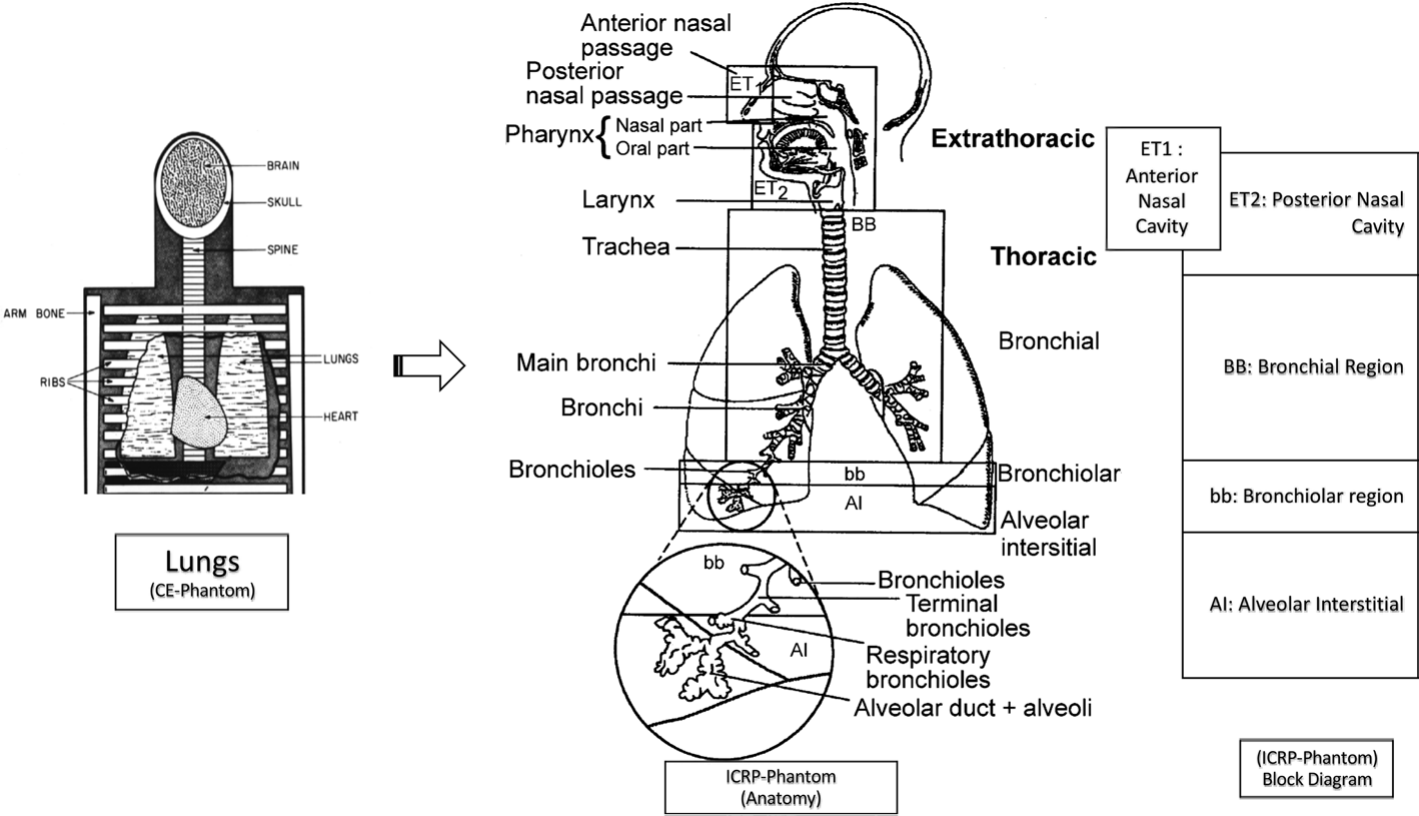
CE to ICRP transition for respiratory tract phantom geometry of source organs (figure adapted from ICRP publication 66 [11] and [6])

**Fig. 2:**
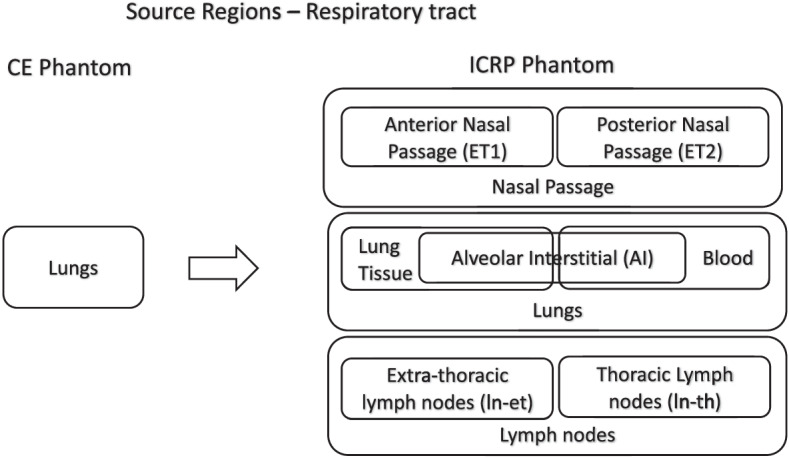
CE respiratory tract source region correspondence to simplified ICRP respiratory tract model for applications in diagnosis and therapy

The updated respiratory tract phantom geometry divides the respiratory tract into several individual source tissue regions into which inhaled radionuclides may deposit. Figure 1 shows the transition from source regions defined in the CE phantom to the morphological phantom developed by the ICRP. The regions of interest for nuclear medicine can be narrowed down to a subset of the regions constituting each section mentioned above. Figure 2 depicts the CE phantom lung regions and the corresponding regions in the ICRP phantom that are relevant to nuclear medicine. The figure was generated using the idea of Venn diagrams to illustrate overlapping regions. As seen by comparing the information on the models in Figs. 1 and 2, these are a subset of the comprehensive list of all source regions provided in the ICRP phantom (Appendix, Table 5).

There are three regions in the ICRP phantom that are coincident with “lungs” as described in the CE phantom. These ICRP phantom regions are listed as: “lungs,” “alveolar interstitial (AI),” and “lung tissue.”

**Fig. 3:**
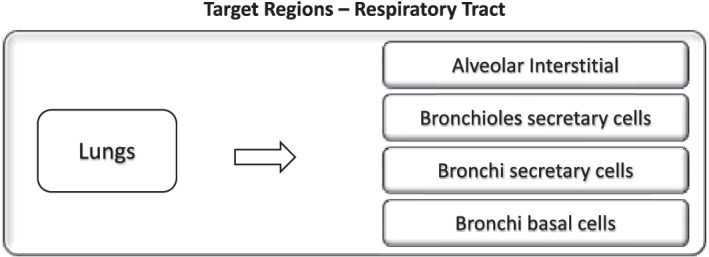
CE to ICRP phantom correspondence (target tissues) for respiratory tract

The region listed as “lung tissue” corresponds to all soft tissues within the lungs (e.g., lung parenchyma) but exclusive of all blood within the lungs (pulmonary arteries, capillaries, and veins). In contrast, the ICRP region listed as “lungs” includes both the lung tissue and its blood content. This distinction is relevant only for biokinetic modeling; in terms of S values, these two source regions are identical. Studies cited in ICRP Publication 66 suggest that blood represents about 58% of the mass of the lungs. The “AI” region is defined as the tissue region supporting the terminal bronchioles. It corresponds to the subregion of the lungs where oxygen and carbon dioxide exchange between the air in the lungs and the blood gases take place. This region is particularly relevant to dosimetry associated with inhaled radioactive particles or potentially to radioactive gas released from the circulation and thus a potential critical target region for emissions originating in adjacent source regions. The nasal passage wall is also associated with inhaled radioactive particles. The nasal passage is comprised of six regions; not all six regions are relevant in radiopharmaceutical therapy. Figure 3 represents the most appropriate target tissue mapping from the CE phantom to the ICRP phantom. The secretory and basal cells are the relevant targets for radiogenic lung cancer and thus they are used by the ICRP in their computation of “lung dose” for radioprotection. For RPT, however, a lung target most representative of the risk of lung fibrosis (or other deterministic effect) is more appropriate. The best ICRP target here would be “lung tissue” which, as noted above, corresponds to the lung parenchyma.

### Alimentary tract

**Fig. 4:**
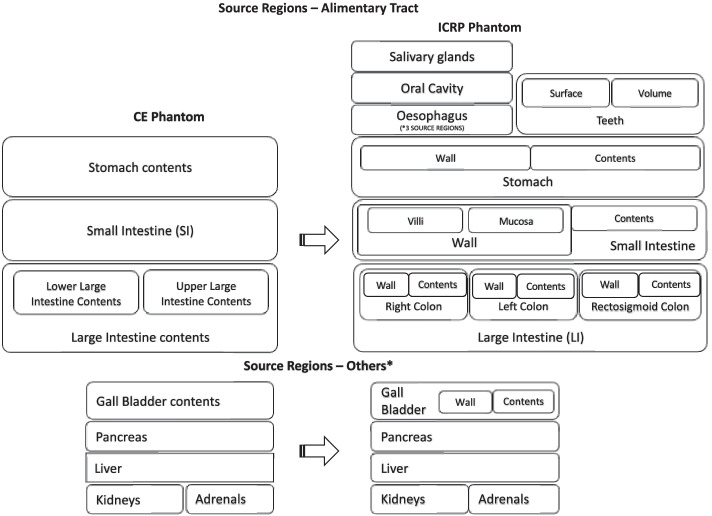
CE to ICRP phantom correspondence (source regions) for the alimentary tract. *Organs which indirectly contribute to the functioning of the alimentary tract

The source regions in the alimentary tract are also differentiated to more explicitly identify tissues that encounter ingested activity (or activity that was inhaled and transferred to the alimentary tract via mucociliary clearance mechanisms). Numerous additional source regions have been defined as a result of alimentary tract phantom development; several of these tissues are not easily sampled either directly or by nuclear medicine imaging, but were included by the ICRP because they are part of their series of radionuclide biokinetic models [12]. This includes tissues in contact with activity due to subsequent transport from the GI tract to blood and organ secretions. Figure 4 shows the evolution of the alimentary tract model from the eight source regions in the CE model to the current ICRP model. In the CE phantom, the S values were calculated using Monte Carlo simulation for radionuclide disintegrations occurring only within organ contents. For the small intestine, however, there is no geometric separation between the SI wall and SI content in the CE phantoms. By contrast, the ICRP Monte Carlo simulations were performed for disintegrations occurring within both the GI tract walls (specifically their mucosal layer) and the contents of the GI tract lumen. Furthermore, the regions are redefined in the ICRP phantom so that the large intestine (LI) is no longer divided into upper large intestine (ULI) and lower large intestine (LLI). Instead, these regions are now redefined as left colon (LC), right colon (RC), and rectosigmoid colon (RS) (Fig. 4), a division that better matches experimental data on content transit times.

The ICRP’s alimentary tract model of Publication 100 [12], which is implemented in the Publication 110 adult phantoms, has additional source regions including the esophagus, teeth, oral cavity, and salivary glands. A portion of the activity in the oral cavity may also be apportioned to oral mucosa. The “salivary glands” source region is comprised of six anatomical regions—the left and right parotid, submandibular, and sublingual glands.

The esophageal wall, oral cavity mucosal lining, teeth surface, and salivary glands are the relevant source or target tissues for absorbed dose calculations in nuclear medicine therapy and diagnosis.

The colon (or large intestines in the CE nomenclature) regions that are defined in the ICRP phantom differ from those in the CE phantom. To transition from the CE to the ICRP phantom, the total activity (ULI + LLI) obtained from the CE phantom is apportioned to the three coincident regions based on their relative masses in the ICRP model. Some other common source and target regions in both the phantoms as shown in Fig. 4 are the kidneys, pancreas, liver, gall bladder, and adrenals. 

Table 1 lists the mass and factors required for an equivalent mapping. Assuming a uniform distribution of activity throughout the colon, the mapping factor times the time integrated activity (TIA) or time integrated activity coefficient (TIAC) in the large intestines gives the TIA or TIAC in the corresponding ICRP source regions.

**Table 1:** Mapping factor to transition from CE to ICRP model

CE source regions	ICRP source region	Acronym	Mass (g)	Mapping factor
ULI (contents) + LLI (contents)	Right colon (contents)	RC-cont	150	0.5
	Left colon (contents)	LC-cont	75	0.25
	Rectosigmoid (contents)	RS-cont	75	0.25
ULI (wall) + LLI (wall)	Right colon (wall)	RC-wall	186.24	0.41
	Left colon (wall)	LC-wall	186.24	0.41
	Rectosigmoid (wall)	RS-wall	86.91	0.18

**Fig. 5:**
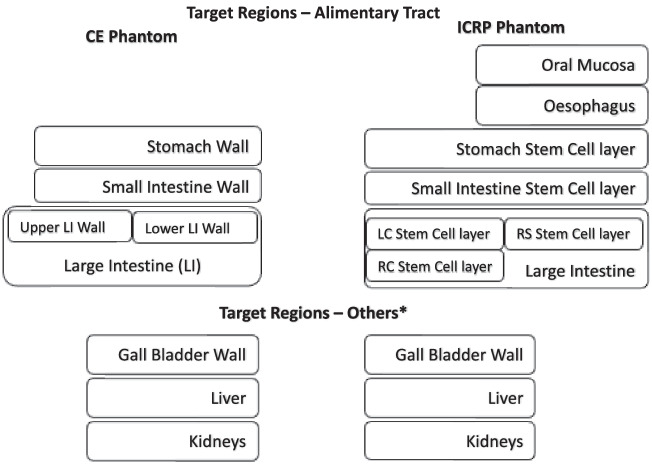
CE to ICRP phantom correspondence (target regions) for the alimentary tract. *Organs which indirectly contribute to the functioning of the alimentary tract

The ICRP phantom alimentary tract includes target regions deemed important in their impact on overall tissue response to radiation. These include the oral mucosa, and the stem cell layers within the mucosal layers of the stomach, small intestine, and large intestine walls. Figure 5 illustrates the corresponding target regions for the CE and ICRP phantoms. In addition to the regions displayed in the figure, ICRP phantom includes the tongue, tonsils, and salivary glands.

### Skeletal tissues

The active marrow, inactive marrow, trabecular and cortical bone are included as source regions in the ICRP phantom’s skeletal system representation. The active marrow is the only marrow source region considered in the original CE phantom.

The definition of bone endosteum has evolved over time. In the ICRP Publication70 model [13], bone endosteum was defined as a single cell layer (10 microns in thickness) along all surfaces of bone trabeculae in trabecular bone, and along the surfaces of the Haversian canals of cortical bone.

With the development of the ICRP 110 adult phantoms, the ICRP changed this target tissue (for radiogenic bone cancer risk [14]) to include a 50-micron region of bone marrow along the surfaces of the bone trabeculae, and a 50-micron layer of marrow along the inner bone shafts of the long bones. Cortical bone is no longer considered to house “endosteum” and thus is only a radiation source region and consequently is no longer a component of the endosteum target region.

**Fig. 6:**
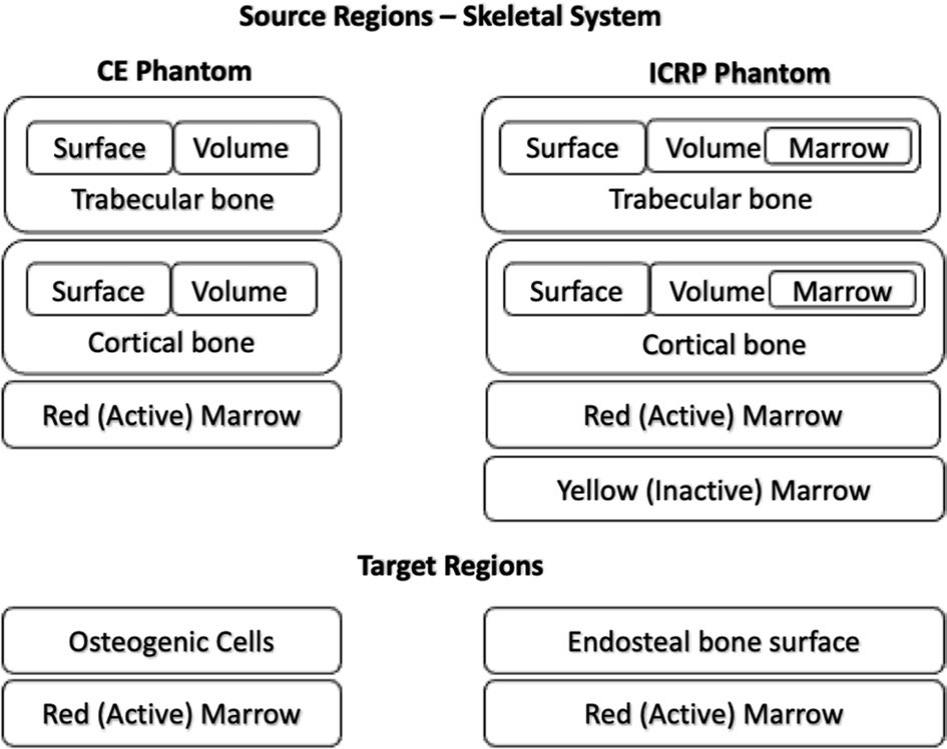
CE to ICRP phantom correspondence in skeletal system

To summarize, the endosteum layer in the ICRP phantom and the osteogenic layer in the CE phantom are the corresponding target regions for the skeletal system. The active marrow is another target region common to both the CE and ICRP phantoms. Both phantoms have an endosteum as a target—but this target is anatomically/histologically different in the two phantoms (Fig. 6).

### Other source and target regions

The spleen, thymus, thyroid, ovaries, testes, uterus, brain, breast, and heart are common as source regions in both the ICRP and CE phantoms. In the CE phantom, the heart was divided into its contents and wall. The ICRP phantom includes the heart wall as a source region; it does not have an explicit heart contents source region since total body blood is included as a distinct source region [9]. The lens of the eye, pituitary gland, tonsils, prostate, salivary glands (including the parotid, submandibular and sublingual), teeth, oral cavity, adipose tissue, and the ureters are new and additional source regions defined within the ICRP adult phantom. Some of these are important in radionuclide therapy dosimetry.

Similarly, there are a few other common target regions included in both models; these are listed in the Appendix. There exist additional target regions corresponding to the additional source tissue in the ICRP adult phantom resulting in a total of 43 target regions.

The CE phantom has a “residual soft tissue (RST)” region corresponding to interior regions of the phantoms that are not already taken by other organs within the body. This RST region is used as a surrogate for “muscle” as a source region and as a target region. In the ICRP phantoms, however, explicit geometric models for both “muscle” and “adipose” tissue are defined; there are no undefined regions in the ICRP phantoms, as there are in the CE phantoms. RST in the CE phantom would correspond to both muscle and adipose tissue in the ICRP phantoms. ICRP categorizes 41 source tissue as “other tissue source” regions which are potential candidates for inclusion in whole body reminder calculation usually performed for RPT dosimetry applications.

### Target mass differences

Target mass differences will impact the absorbed dose comparison. Table 2 summarizes the target region masses for the two different phantoms. The GI tract targets in the CE phantom were entire organ walls, while in the ICRP phantoms they are stem cell layers embedded at depth within only the outer mucosal layer of the GI tract walls.

**Table 2:** CE phantom to ICRP phantom mapping for major alimentary tract target tissues

CE target region	Mass (g)	ICRP target region	Mass (g)
Lower LI wall	1.67E + 02	Right colon stem cell layer	1.35E + 00
Upper LI wall	2.20E + 02	Left colon stem cell layer	1.26E + 00
		Rectosigmoid stem cell layer	7.59E − 01
Stomach wall	1.58E + 02	Stomach stem cell layer	6.16E − 01
Small intestine	6.77E + 02	Small intestine stem cell layer	3.71E + 00
Osteogenic cells	1.08E + 02	Endosteal bone surface	5.80E + 02

### Blood as source region

To facilitate biokinetic modeling wherein a central blood pool is a distinct physiologic compartment, the ICRP phantom considers blood as a distinct source region. Accordingly, source regions correspond to and are assigned the mass of tissue parenchyma. Activity in blood also contributes to the total organ activity, and thus the organ self-doses and cross-doses. Source region activity values estimated from nuclear medicine imaging will include activity in the whole organ (blood + parenchyma). Using a measured blood value directly would lead to double counting of the blood in such regions since source region activity measured from the images includes both the radiopharmaceutical activity in the organ parenchyma as well as the radiopharmaceutical activity within the blood pool of that same organ. This can be avoided by subtracting the fraction of total body blood activity that is localized within the blood pool of the organ of interest (based upon reference blood distribution models [15]). The CE phantom does not include blood as a source organ except for (1) the heart contents region and (2) the major inter-organ veins and arteries. There are no explicit models of intra-organ blood vessels in the organs of the Publication 110 reference voxel phantoms.

### Suggested limitations on the simultaneous use of source regions

The ICRP phantom includes several source regions that overlap to allow modeling of radionuclides that distribute by different mechanisms. Some of these source regions should not be used together as they overlap. Other structures, including volume and surface sources for some regions, would not normally be used together if the data came from imaging or tissue sampling, but might be based on modeling. Table 3 gives a list of the cases where such limitations make sense.

**Table 3:** Suggested use on simultaneous use of source regions

Source region A^c^	Source region B^c^	Simultaneous use of A and B
Lung-tis	Lungs	Prohibited^a^
Remainder	Blood	Prohibited
Lung-tis	AI, brchiole-b, brchiole-q, brchiole	Prohibited
Lungs	AI, brchiole-b, brchiole-q, brchiole	Prohibited
C-bone-v	C-bone-s	Unusual^b^
ET1-wall	ET1-sur	Unusual
ET2-wall	ET2-bnd, ET2-seq, ET2-sur	Unusual
Esophag-w	Esophag-s, Esophag-f	Unusual
LC-wall	LC-mucosa	Prohibited
RC-wall	RC-mucosa	Prohibited
RS-wall	RS-mucosa	Prohibited
SI-wall	SI-villi, SI-mucosa	Prohibited
ST-wall	ST-mucosa	Prohibited
Teeth-v	Teeth-s	Unusual
T-bone-v	T-bone-s	Unusual
C-marrow	R-marrow	Prohibited
C-marrow	Y-marrow	Prohibited
T-marrow	R-marrow	Prohibited
T-marrow	Y-marrow	Prohibited

^a^Prohibited indicates that the source region A overlaps with the source or sources in source region B and should not be used together

^b^Unusual indicates that the sources do not overlap, but it would be difficult to measure the activity in the sources from imaging and would likely require modeling

^c^Abbreviations of source regions listed are given in the Appendix (Table 5)

## Discussion

**Fig. 7:**
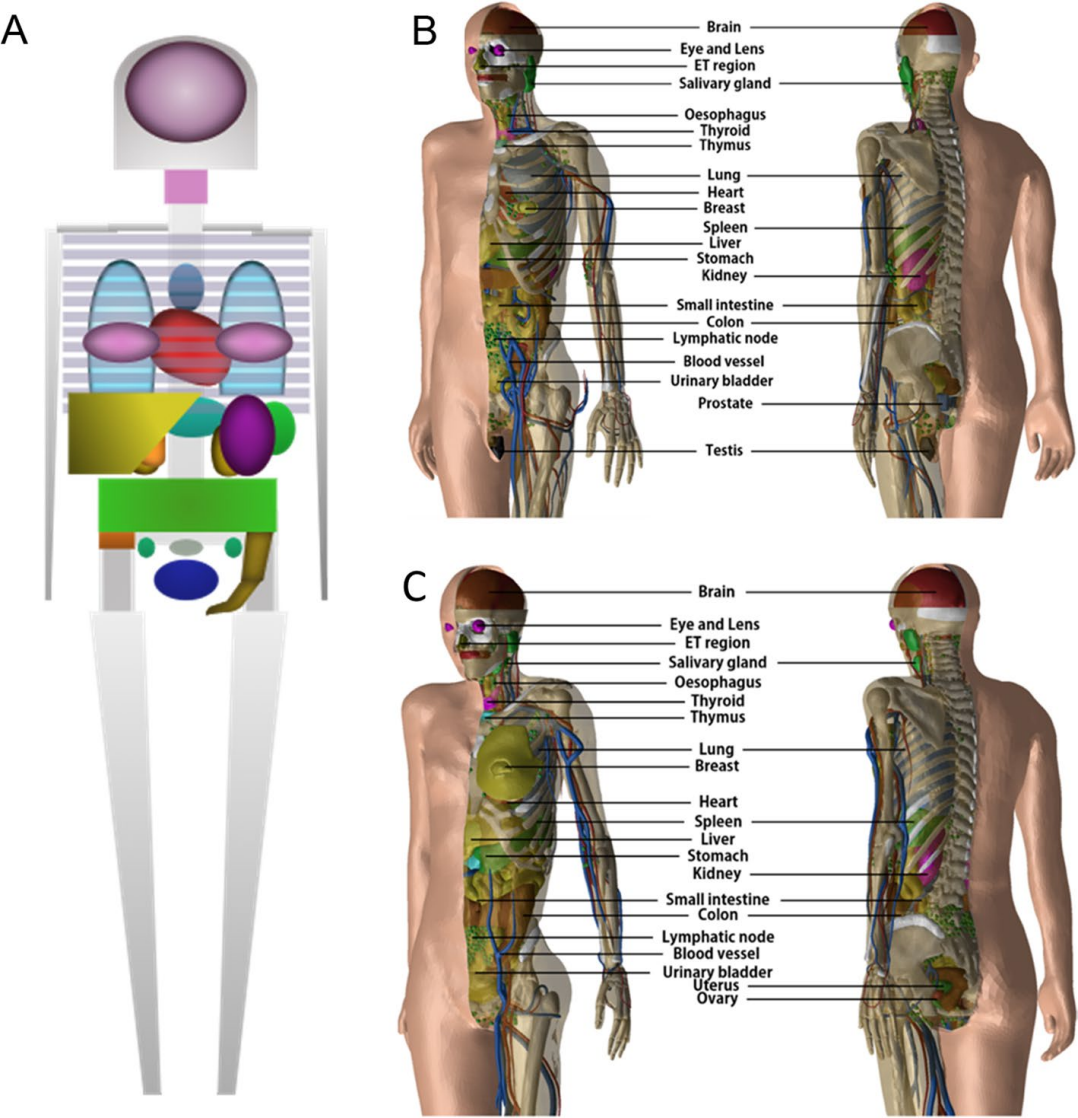
Representation of **A** the CE phantom (stylized model using [2] as reference) and **B** the mesh version of ICRP 110 voxelized reference adult male phantom **C** the mesh version of ICRP 110 voxelized reference adult female phantom [16]

The work above is important in the context of the need for standardized, well-validated quantitative imaging and dosimetry techniques for RPT. If, for a given input data set, the results of a dosimetry calculation are not the same everywhere (i.e., with the same level of accuracy), the argument for adopting RPT dosimetry and treatment planning anywhere is difficult to make. The benefits of dosimetry-driven RPT treatment planning will be difficult if not impossible to demonstrate if absorbed doses and corresponding patient outcomes are not comparable wherever RPT is implemented. At present, dosimetry calculations for radiopharmaceuticals are performed, for the most part, with S values derived from absorbed fractions calculated using phantoms developed in the late 1970s through the 1980s (i.e., the Cristy–Eckerman phantoms). The absorbed fractions calculated using these phantoms focus primarily on photon transport; the dose contribution from electrons was set either to 1 (organ self-dose) or zero (cross-organ dose). The absorbed dose contribution to the wall from contents was set to 0.5 times the content of the self-irradiation absorbed dose. In 2017, the ICRP released new realistic phantoms [Fig. 7]. These were generated by segmenting patient CT scans. The specific absorbed fractions calculated using these new phantom geometries include both electron and alpha-particle transport (using stylized models for selected tissue). Intra-skeletal tissue dosimetry in the ICRP SAFs is now based upon 3D radiation transport calculations in both CT-based macroscale and microCT-based microscale models of skeletal tissues [18]. As Fig. 7 shows, there are substantial differences in anatomical realism between the two phantom types.

## Conclusion

Despite the detailed anatomical representation and improved accuracy, calculations using these new phantoms have not been widely adopted. The comparisons provided in this work are intended to help transition the field to these new models, which are anatomically and computationally accurate. As a first step, we established a correspondence between CE phantom and ICRP phantom source/target tissue nomenclature (Fig. 1–6). We have also endeavored to identify tissue mass differences and use these to provide guidance on how to apportion TIA/TIAC originally derived for CE Phantom anatomic geometry (Table 1).

## Data Availability

Not applicable.

## Appendix

See Tables 4, 5 and 6.

**Table 4:** Source and target tissue masses described in OLINDA and CE phantom for reference adult male phantom

Reference adult male phantom organ masses (kg)		
Source and target regions^1^	Masses listed in OLINDA software	CE phantom paper
Adrenals	1.63E − 02	1.63E − 02
Bone, cort	4.00E + 00	4.00E + 00
Bone, trab	1.00E + 00	1.00E + 00
Brain	1.42E + 00	1.42E + 00
Breasts	3.51E − 01	3.51E − 01
Gallbladder wall	1.05E − 02	1.05E − 02
Gall bladder contents	n/a	5.57E − 02
LLI wall	1.67E − 01	1.67E − 01
LLI contents	n/a	1.43E − 01
ULI walls	2.20E − 01	2.20E − 01
ULI contents	n/a	2.32E − 01
Small intestine	6.77E − 01	1.10E + 00
Stomach wall	1.58E − 01	1.53E − 01
Stomach contents	n/a	2.60E − 01
Heart contents	n/a	4.54E − 01
Heart wall	3.16E − 01	3.16E − 01
Kidneys	2.99E − 01	2.99E − 01
Liver	1.91E + 00	1.91E + 00
Lungs	1.00E + 00	1.00E + 00
Muscle	2.80E + 01	5.18E + 01
Osteogenic cells	1.20E − 01	1.08E − 01*
Ovaries	0.00E + 00	0.00E + 00
Pancreas	9.43E − 02	9.43E − 02
Red marrow	1.12E + 00	1.12E + 00
Spleen	1.83E − 01	1.83E − 01
Testes	3.91E − 02	3.91E − 02
Thymus	2.00E − 02	2.09E − 02
Thyroid	2.07E − 02	2.07E − 02
Urinary bladder wall	4.76E − 02	4.76E − 02
Urinary bladder contents	n/a	2.11E − 01
Uterus/uterine wall	0.00E + 00	0.00E + 00

*****CE phantom does not list endosteal cell mass; the value listed was obtained from reference [17]

**Table 5:** ICRP 110 adult male and female reference phantom source tissue masses*

Source region	ICRP 110 ref adult male tissue mass (kg)	ICRP 110 ref adult female tissue mass (kg)	Acronym
Adipose	1.751E + 01	2.176E + 01	Adipose
*Adrenals*	*1.736E − 02*	*1.546E − 02*	*Adrenals*
Alveolar-interstitial	1.100E + 00	9.000E − 01	AI
Blood	5.600E + 00	4.100E + 00	Blood
*Brain*	*1.517E* + *00*	*1.349E* + *00*	*Brain*
Bronchiolar surface	0.000E + 00	0.000E + 00	Brchiole
Bronchiolar bound region	4.891E − 03	4.703E − 03	Brchiole-b
Bronchiolar sequestered region	1.252E − 03	1.204E − 03	Brchiole-q
*Breast*	*2.617E − 02*	*5.154E − 01*	*Breast*
Bronchial surface	0.000E + 00	0.000E + 00	Bronchi
Bronchial bound region	1.727E − 03	1.552E − 03	Bronchi-b
Bronchial sequestered region	2.918E − 04	2.622E − 04	Bronchi-q
Cartilage	1.156E + 00	9.410E − 01	Cartilage
Cortical bone surfaces	0.000E + 00	0.000E + 00	C-bone-S
Cortical bone volumes	4.445E + 00	3.233E + 00	C-bone-V
Cortical marrow	2.790E − 01	2.580E − 01	C-marrow
ET1 surface	0.000E + 00	0.000E + 00	ET1-sur
ET1 wall	3.991E − 03	2.778E − 03	ET1-wall
ET2 bound region	2.472E − 03	2.137E − 03	ET2-bnd
ET2 sequestered region	4.504E − 04	3.894E − 04	ET2-seq
ET2 surface	0.000E + 00	0.000E + 00	ET2-sur
ET2 wall	3.991E − 03	2.778E − 03	ET2-wall
Eye Lens	4.000E − 04	4.000E − 04	Eye-lens
*Gall bladder content*	*5.800E − 02*	*4.800E − 02*	*GB-cont*
Gall bladder wall	1.047E − 02	8.246E − 03	GB-wall
Heart wall	3.860E − 01	2.910E − 01	Ht-wall
*Kidneys*	*4.220E − 01*	*3.570E − 01*	*Kidneys*
Left colon content	7.500E − 02	8.000E − 02	LC-cont
Left colon mucosa	1.875E − 02	1.726E − 02	LC-mucosa
Left colon wall	1.862E − 01	1.714E − 01	LC-wall
*Liver*	*2.360E* + *00*	*1.810E* + *00*	*Liver*
Lymph nodes—extrathoracic	1.595E − 02	1.270E − 02	LN-ET
Lymph nodes—systemic	1.577E − 01	1.255E − 01	LN-Sys
Lymph nodes—thoracic	1.595E − 02	1.270E − 02	LN-Th
Lungs (with blood)	1.200E + 00	9.500E − 01	Lungs
*Lung Tissue*	*1.200E* + *00*	*9.500E − 01*	*Lung-Tis*
Muscle	2.978E + 01	1.793E + 01	Muscle
Oral cavity	0.000E + 00	0.000E + 00	O-cavity
Esophagus—fast	0.000E + 00	0.000E + 00	Esophag-f
Esophagus—slow	0.000E + 00	0.000E + 00	Esophag-s
Esophagus wall	4.904E − 02	4.129E − 02	Esophag-w
Oral mucosa	3.583E − 02	2.245E − 02	O-mucosa
*Ovaries*	*0.000E* + *00*	*1.264E − 02*	*Ovaries*
*Pancreas*	*1.736E − 01*	*1.446E − 01*	*Pancreas*
Pituitary gland	6.280E − 04	6.180E − 04	P-gland
Prostate	1.780E − 02	0.000E + 00	Prostate
Right colon content	1.500E − 01	1.600E − 01	RC-cont
Right colon mucosa	2.010E − 02	1.773E − 02	RC-mucosa
Right colon wall	1.862E − 01	1.714E − 01	RC-wall
*Red marrow*	*1.394E* + *00*	*1.064E* + *00*	*R-marrow*
Rectosigmoid colon content	7.500E − 02	8.000E − 02	RS-cont
Rectosigmoid colon mucosa	1.128E − 02	1.039E − 02	RS-mucosa
Rectosigmoid colon wall	8.691E − 02	8.273E − 02	RS-wall
Respiratory tract air	0.000E + 00	0.000E + 00	RT-air
Salivary glands	8.898E − 02	7.215E − 02	S-glands
Small intestine content	3.500E − 01	2.800E − 01	SI-cont
Small intestine mucosa	3.696E − 02	3.432E − 02	SI-mucosa
Small intestine villi	1.252E − 02	1.252E − 02	SI-villi
Small intestine wall	8.088E − 01	7.163E − 01	SI-wall
*Skin*	*3.468E* + *00*	*2.423E* + *00*	*Skin*
*Spleen*	*2.284E − 01*	*1.874E − 01*	*Spleen*
*Stomach content*	*2.500E − 01*	*2.300E − 01*	*St-cont*
Stomach mucosa	4.639E − 03	4.639E − 03	St-mucosa
Stomach wall	1.839E − 01	1.652E − 01	St-wall
Trabecular bone surfaces	0.000E + 00	0.000E + 00	T-bone-S
*Trabecular bone volumes*	*1.167E* + *00*	*8.492E − 01*	*T-bone-V*
Teeth surfaces	0.000E + 00	0.000E + 00	Teeth-S
Teeth volumes	5.000E − 02	4.000E − 02	Teeth-V
*Testes*	*3.724E − 02*	*0.000E* + *00*	*Testes*
*Thymus*	*2.617E − 02*	*2.062E − 02*	*Thymus*
*Thyroid*	*2.336E − 02*	*1.946E − 02*	*Thyroid*
Trabecular marrow	3.371E + 00	2.442E + 00	T-marrow
Tongue	7.642E − 02	6.185E − 02	Tongue
Tonsils	3.141E − 03	3.092E − 03	Tonsils
*Urinary bladder content*	*2.000E − 01*	*2.000E − 01*	*UB-cont*
Urinary bladder wall	5.112E − 02	4.082E − 02	UB-wall
Ureters	1.675E − 02	1.546E − 02	Ureters
*Uterus*	*0.000E* + *00*	*8.246E − 02*	*Uterus*
Yellow marrow	2.480E + 00	1.800E + 00	Y-marrow
Totals (all listed tissues):	73.3064824	60.1019016	

*Italicized rows correspond to source regions listed in CE phantom/OLINDA compilation

**Table 6:** Target tissue regions and their masses (kgs) as given in ICRP 133*

Masses inclusive of blood (kg)			
Target region	Adult male	Adult female	Acronym
Oral Mucosa	3.58E − 02	2.25E − 02	O-mucosa
Esophagus	9.50E − 05	8.80E − 05	Esophagus
Stomach stem cell layer	6.16E − 04	6.16E − 04	St-stem
Small intestine stem cell layer	3.71E − 03	3.45E − 03	SI-stem
Right colon stem cell layer	1.35E − 03	1.19E − 03	RC-stem
Left colon stem cell layer	1.26E − 03	1.16E − 03	LC-stem
Rectosigmoid Colon stem cell layer	7.59E − 04	6.99E − 04	RS-stem
ET1 basal cells	2.00E − 05	1.70E − 05	ET1-bas
ET2 basal cells	4.50E − 04	3.90E − 04	ET2-bas
Extrathoracic lymph nodes	1.60E − 02	1.27E − 02	LN-ET
Bronchi basal cells	4.30E − 04	3.90E − 04	Bronch-bas
Bronchi secretary cells	8.60E − 04	7.80E − 04	Bronch-sec
Bronchioles secretary cells	1.90E − 03	1.90E − 03	Bchiol-sec
Alveolar interstitial	1.10E + 00	9.04E − 01	AI
Thoracic lymph nodes	1.60E − 02	1.27E − 02	LN-Th
*Red (active) marrow*	*1.39E* + *00*	*1.06E* + *00*	*R-marrow*
*Endosteal cells*	*5.80E − 01*	*4.33E − 01*	*Endost-BS*
*Brain*	*1.52E* + *00*	*1.35E* + *00*	*Brain*
Lens of the eye	4.00E − 04	4.00E − 04	Eye-lens
Pituitary gland	6.28E − 04	6.18E − 04	P-gland
Tongue	7.64E − 02	6.19E − 02	Tongue
Tonsils	3.14E − 03	3.09E − 03	Tonsils
Salivary glands	8.90E − 02	7.22E − 02	S-glands
*Thyroid*	*2.34E − 02*	*1.95E − 02*	*Thyroid*
*Breast*	*2.62E − 02*	*5.15E − 01*	*Breast*
*Thymus*	*2.62E − 02*	*2.06E − 02*	*Thymus*
*Heart wall*	*3.86E − 01*	*2.91E − 01*	*Ht-wall*
*Adrenals*	*1.74E − 02*	*1.55E − 02*	*Adrenals*
*Liver*	*2.36E* + *00*	*1.81E* + *00*	*Liver*
*Pancreas*	*1.74E − 01*	*1.45E − 01*	*Pancreas*
*Kidneys*	*4.22E − 01*	*3.57E − 01*	*Kidneys*
*Spleen*	*2.284E − 01*	*1.87E − 01*	*Spleen*
*Gall bladder wall*	*1.05E − 02*	*8.25E − 03*	*GB-wall*
Ureters	1.68E − 02	1.55E − 02	Ureters
*Urinary bladder wall*	*5.11E − 02*	*4.08E − 02*	*UB-wall*
*Ovaries*	*0.00E* + *00*	*1.26E − 02*	*Ovaries*
*Testes*	*3.72E − 02*	*0.00E* + *00*	*Testes*
Prostate	1.78E − 02	0.00E + 00	Prostate
*Uterus*	*0.00E* + *00*	*8.25E − 02*	*Uterus*
Systemic lymph nodes	1.58E − 01	1.26E − 01	LN-Sys
Skin	3.47E + 00	2.42E + 00	Skin
Adipose tissue	1.75E + 01	2.18E + 01	Adipose
*Muscle*	*2.98E* + *01*	*1.79E* + *01*	*Muscle*

*Italicized rows correspond to target tissue regions listed in CE phantom/OLINDA compilation
